# Pitfalls in website information on dietary sodium restriction

**DOI:** 10.1111/jch.14587

**Published:** 2022-10-09

**Authors:** Decio Armanini, Luciana Bordin, Jacopo Manso, Marco Boscaro, Chiara Sabbadin

**Affiliations:** ^1^ Department of Medicine–Endocrinology University of Padova Padova Italy; ^2^ Department of Molecular Medicine‐Biological Chemistry University of Padova Padova Italy

## 1 INTRODUCTION

The FDA recommends that we should not have more than 2 g of sodium per day (one teaspoon).^1^ This rate represents a level of sodium considered safe and adequate for the general population of adults, including children aged 15 years and over and pregnant or lactating women. Only for infants and children sodium intake should be reduced, based on their age and energy requirement: 0.2 g/day is proposed for infants aged 7–11 months, 1.1 g/day for children aged 1–3 years, 1.3 g/day for children aged 4–6 years and 1.7 g/day for children aged 7–15 years. However, the average intake of sodium for most Americans is more than 3 g per day and similar results are reported in oriental countries, where salt is added to many foods.^2^ The purpose of these recommendations is to progressively reduce the daily salt intake of Americans improving their quality of life and increasing lifespan itself. The improvement will be reflected especially in the reduction of hypertension and cardiovascular diseases. Currently, 40% of the adult population and 10% of children are hypertensive. Hypertension involves genetic, racial, and quality‐of‐life factors.

In the recent study of Hussain and coll.,^3^ the authors conclude that most popular websites provide either information or guidance on dietary sodium reduction, but rarely both. The two most relevant tools that evaluate information on sites dealing with sodium restriction are the DISCERN^4^ and the JHU‐SALT.^3^ The Authors conclude that consumers seeking information and guidance online will find that most easily accessible websites offer accurate but limited information and provide insufficient guidance on how to lower sodium intake.

The doctor‐patient relationship very often is conditioned by the Web. Currently, people search for news of some possible disease or nutritional recommendations. Even the choice of a specialist is often based on web searches. Often patients have no idea of their salt intake and might seek information about salt intake either because of their interest in healthier living or because they have hypertension or another chronic disease. Many sites report that salt restriction is critical for maintaining regular blood pressure and reducing cardiovascular risk and suggest that everyone should follow a low‐salt diet. Unfortunately, those seeking information on the Internet lack knowledge of the complex mechanisms of water and electrolyte regulation and perhaps follow incorrect instructions for their case. Often the low‐sodium diet is recommended on sites to normotensive patients, or it is not explained that in all cases an evaluation of the cause of possible alterations should be sought before taking unnecessary and even harmful diets and supplements. Each pathology must be considered in relation to possible implications in sodium secretion or excretion and to associated current therapies, which are often complex and include medications that clearly affect hydro‐electrolyte balance. This demonstrates how an inexperienced or out‐of‐date patient on this topic might be misled when examining websites that recommend salt reduction. The patient's history should always investigate possible incorrect sources of information, especially on the current topic about salt or sodium intake. The same applies to water intake that is strictly related to salt.

### 1.1 Regulation of sodium and volume

**FIGURE 1:**
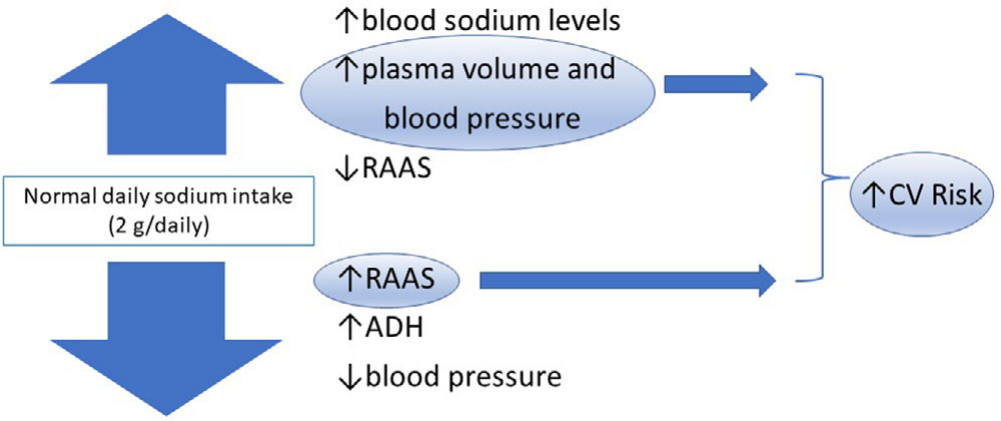
Relationship between sodium intake and aldosterone in the regulation of blood pressure. ADH, antidiuretic hormone; CV, cardiovascular, RAAS, renin angiotensin aldosterone system.

It is known that sodium is the basic element in the body and is regulated by various factors, such as water intake, activity of the renin‐angiotensin aldosterone system (RAAS), antidiuretic hormone (ADH), natriuretic hormones, cortisol, body mass index, insulin resistance, and many other factors.^5^ Any alteration in sodium and volume must be compensated by regulatory mechanisms, as happens throughout all the endocrine system. An exaggerated intake of water can dilute sodium but also has a suppressing action on renin production and thus aldosterone, accentuating fluid excretion to restore a physiological balance. The opposite occurs in cases of fluid loss or reduced water intake as frequently happens in the elderly. This condition leads to a hemoconcentration and stimulates the RAAS and ADH, both to conserve outputs and to improve water and electrolyte balance (Figure 1).

Searching websites for information about low‐sodium foods and diets could be dangerous, leading to an activation of the RAAS. It is known that aldosterone has a potent inflammatory and fibrotic action when produced in excess. A similar situation has been demonstrated in patients taking furosemide and thiazides, which on the one hand reduce blood pressure, but on the other hand accentuate the inflammatory state and cardiovascular risk.^6^ Therefore, it is always recommended to combine a potassium‐sparing drug such as spironolactone and its derivatives.^7^ These considerations show how important the intervention of the specialist is in deciding on both diet and medication needed in the treatment of hypertension.

### 1.2 Estimation of sodium intake

Estimation of salt intake is very complex and current studies give conflicting results, especially because the actual criteria are still inadequate and involve many factors that are difficult for the patient to assess and must be explained and evaluated by the specialist. Some studies have considered sodium intake either by questionnaires or by assessment of 24‐h sodium excretion, while others have assessed sodium excretion in a spot urine sample.^8^ All these assessments can be a source of error both for eventual treatments and for genetic differences in the body's response to salt intake. The nature and quality of nutrient intake ascertainment are diverse. Some foods, consumed especially by young people, contain exaggerated amounts of salt, such as sandwiches, pancakes, energy drinks, chips, soup. Self‐reported measures may be essential for some purposes but intrinsically suffer from both random errors and systematic biases. Biomarkers of intake, which are more objective, can replace self‐report for some purposes, but have only been developed for a few nutrients. Currently, no one single approach accurately measures dietary intakes in a comprehensive manner for all nutrients. We believe that the web information cannot be accurate because the sites do not know the history and eating habits of each individual person seeking information about salt intake and possible correlated diseases.

## 2 CONCLUSIONS

Through this commentary, we want to point out that JCH readers, who are doctors involved in hypertension, need to understand that often patients before going to the doctor inform themselves by looking for websites that explain the issue. The problem is that the sites may not have information about the specific problem of the readers and therefore they propose standard diets and therapy that in specific cases could be harmful. Even some doctors write on the web their considerations related to the issue and in each case, the conclusions cannot always be correct.

The study of Hussain and coll.^3^ is interesting since it highlights this issue. It would be important that the conclusions of this study could be available not only to JCH readers who may be experts on the issue, but also to the media to urge them to always contact specialists before deciding whether to undertake a low‐salt diet. The most important Scientific Societies involved in hypertension and cardiovascular disorders should periodically share simple and clear information about some burning issues, such as salt intake, licorice abuse, and endocrine causes of hypertension. Family doctors need to know the patients' sources of information and they should encourage patients to consider only these official websites.

The physician's role is therefore critical in order to discuss and alert patients to the positive effects and risks of a low‐sodium diet. Changes of life‐style and nutritional habits should always be considered according to patients’ history and concomitant disorders. It is known that some populations abuse salt and other populations are predisposed to hypervolemia such as African American patients in whom low‐renin essential hypertension is easily found.^9^ Genetic predisposition to hypertension and alterations of parameters linked to plasma volume regulation must always be considered by physicians to give appropriate advice to each individual case. The patient's history should always include an assessment of inappropriate diets, exaggerated fluid intake, or unnecessary salt reduction. Such data are also important to interpret any altered laboratory data.

## CONFLICT OF INTEREST

The authors have no competing interests.
